# Association of parent-child health parameters and lifestyle habits - the “epi-family health” longitudinal study protocol

**DOI:** 10.1186/s13690-024-01311-7

**Published:** 2024-06-11

**Authors:** Diego Giulliano Destro Christofaro, William Rodrigues Tebar, Claudiele Carla Marques da Silva, Bruna Thamyres Ciccotti Saraiva, Amanda Barbosa Santos, Ewerton Pegorelli Antunes, Enrique Gervazoni Ferreira Leite, Isabella Cristina Leoci, Victor Spiandor Beretta, Gerson Ferrari, Jorge Mota, Luiz Carlos Marques Vanderlei, Raphael Mendes Ritti-Dias

**Affiliations:** 1 School of Technology and Sciences, Department of Physical Education, São Paulo State University (Unesp), Rua Roberto Simonsen 305, Zip Code, 19060-900 Presidente Prudente, SP Brazil; 2 Faculty of Health Sciences, Universidad Autónoma de Chile, Providencia, Santiago, Chile; 3 Research Center in Physical Activity, Health and Leisure (CIAFEL), Faculty of Sports, Laboratory for Integrative and Translational Research in Population Health (ITR), University of Porto (FADEUP), Porto, Portugal; 4 Department of Physical Education, Universidade Nove de Julho, Sao Paulo, SP Brazil

**Keywords:** Mother, Father, Son, Lifestyle habits, Physical activity

## Abstract

**Background:**

Lifestyle and habits, cardiovascular risk factors (CRF), bone and mental health, dietary habits, physical activity, among others are developed in childhood and adolescence. Family environment has shown to play an important role in these outcomes. However, whether the parent-child relationship lifestyle habits and health parameters can be influenced by physical activity patterns still unclear. The objective of this study will be to monitor and investigate the associations between lifestyle habits between parents and their children longitudinally, as well as verify whether in more active parents, the possible associations with lifestyle habits are different from those of parents considered less active.

**Methods:**

The sample will consist of parents (father, mother, or both) and their children /adolescents. The participants will be recruited through public call by flyers spread across all the regions of the city and also through social media. The health parameters will include cardiovascular (cardiac autonomic modulation, blood pressure and resting heart rate), bone mineral density, anthropometric indices, handgrip strength, mental health (quality of life, anxiety and depression symptoms and stress), self-reported morbidities and musculoskeletal pain. Lifestyle habits will include physical activity levels, sedentary behavior, sleep parameters, eating patterns, smoking and alcohol consumption. Sociodemographic variables of age, sex, ethnicity and socioeconomic status will be considered as covariates. The follow-up visits of data collection will be scheduled after a period of 12 months from the baseline assessment during every twelve months.

**Discussion:**

The family environment has great potential to determine lifestyle habits in children and adolescents. Based on the results presented in the present study, we hope that health promotion actions can be better designed in the family environment.

**Table 1:** 

Text box 1: Contribution to the literature
• There is limited evidence from longitudinal studies on how parents’ lifestyle habits can influence those of their children, especially in developing countries.
• The present study will analyze the role of physical activity in different domains and intensities in lifestyle habits among parents and their children.
• Our expectation is that health promotion actions can be better directed in the family environment with the future findings of this study.

## Introduction

Childhood and adolescence are important periods in a human being’s life, as many characteristics and lifestyle habits acquired at these stages of life have a higher chance of being carried into adulthood. Among these characteristics and habits, body composition [1], cardiovascular health variables such as blood pressure and resting heart rate [2], eating habits [3], sleep time and quality [4], mental health [5], sedentary behavior [6], physical activity [7], among others.

One factor that has shown to influence the lifestyle habits of children and adolescents is the behavior of their parents. Studies have shown relationships between parents and their children regarding obesity [8], bone health [9], hypertension [10], eating habits [11], mental health [12], sleep [13], sedentary lifestyle [14] and physical activity [15, 16]. Parents generally serve as an example and are the ones who provide social support for important habits such as eating and physical activity.

In relation to this topic, a gap that requires investigation in the existing literature is whether the physical activity levels of parents can impact various domains of their children’s lifestyle and health. Specifically, it is important to explore whether physical activity conducted in different domains (such as occupational physical activity, leisure-time sports, and commuting activities) and intensities (light, moderate, vigorous) can influence these relationships. In addition, the cross-sectional design of previous studies is a limitation in the current literature [10, 13, 15], which prevents the inference of causality, and the information derived in studies from developing countries such as Brazil is another gap, as most of the information available in the literature were obtained in developed countries [8, 17].

Therefore, the objective of this study will be to monitor and investigate the associations between lifestyle habits between parents and their children longitudinally, as well as verify whether in more active parents, the possible associations with lifestyle habits are different from those of parents considered less active. Our hypothesis is that lifestyle characteristics and habits between parents and children are positively associated in more active parents.

## Methods

The present study entitled the EPI-Family Health Study.

This study is an initiative to monitor different health parameters in the family environment. We will assess autonomic and cardiovascular systems (cardiac autonomic modulation, blood pressure and resting heart rate), bone mineral density, anthropometric indices, handgrip strength, mental health (quality of life, anxiety and depression symptoms and stress), self-reported morbidities and musculoskeletal pain outcomes. Lifestyle habits will include physical activity levels, sedentary behavior, sleep quality and time, eating patterns, smoking and alcohol consumption.

The invitation to participate in the study will be made through different means (social media, posters, folders, researchers going to schools). At the end of the evaluations, a didactic feedback will be prepared with the main results and a doubt channel will be made available where participants can be guided. This study will have a longitudinal design and the objective is for reassessments to take place annually. It is noteworthy that the present study is supported by the São Paulo Research Foundation (FAPESP; process: 2022/16437-8).

### Sample

The sample for this longitudinal study will consist of adults and children and adolescents aged 6–17 years. The study will be carried out in the city of Presidente Prudente, located in the southeast region of Brazil and which has approximately 230,000 inhabitants according to information from the Brazilian Institute of Geography and Statistics.

Sample recruitment will take place through flyers spread across the five regions of the city (north, south, east, west and central area) and also through social media (Facebook, Instagram, Whatsapp, radio, TV) and researchers going to schools to extend the invitation. The inclusion criteria will be considered: i) children, adolescents and parents who do not have a pacemaker (as it can influence the results of the assessment of cardiac autonomic modulation, ii) who carry out all the assessments proposed by the study; iii) who have not used any type of caffeinated or alcoholic beverage in a period of 12 h before the assessments.

To calculate the sample, a correlation of 0.31 was used based on the study by Christofaro et al. [15] for the relationship between the physical activity of parents and children, considering a power of 80% and an alpha error of 5%. Anticipating possible sample loss, an additional 10% will be added to this calculation, totaling a minimum number of 176 participants. However, as multiple regressions will be carried out in the present study, the sample size was increased by 20%, totaling a final sample number of 212 participants to be evaluated (106 children and/or adolescents and at least one of their parents to be evaluated). The intention of the present study is to carry out reassessments every twelve months.

### Ethical considerations

The present study was approved by the Ethics and Research Committee of the São Paulo State University (UNESP) (CAAE: 59261422.2.0000.5402). All participants will have to sign the consent form agreeing to participate in the study. Children and adolescents will also sign a consent form agreeing to participate in the study. The present study is registered on the clinicaltrials.gov platform (protocol: NCT06248164).

### Data collection

After sample recruitment has been carried out, data collection will begin. Graduate and undergraduate students will carry out data collection. Before data collection, all students will receive training for assessments in all procedures used in this study (questionnaires, accelerometers, heart rate monitors and DEXA).

Initially, participants will come to the São Paulo State University (UNESP). In this first evaluation, the interview will be carried out through the application of a questionnaire, in which the evaluators will collect data face to face with the participant. They will also carry out an assessment of blood pressure, resting heart rate and cardiac autonomic modulation through heart rate variability and at the end of this assessment the accelerometer will be given to the research participant with explanations and how this instrument should be used. Accelerometer collection procedures are described below:

(i) The individual will receive the accelerometer and one of the evaluators will explain how the device works (how to use and when to remove it) and will also leave a small manual with information about the device; (ii) Based on the cell phone number entered in the registration form of each subject who has the accelerometer, messages will be sent via SMS and/or WhatsApp reminding the study participant to use the accelerometer. If the subject only has a home telephone, the evaluator responsible for that subject will make a call reminding the subject to put on the device; (iii) On a pre-scheduled date, the participant will come to UNESP, deliver the accelerometer and carry out the final assessment, consisting of anthropometry (weight, height, waist circumference) and measurement of body composition using DEXA.

### Assessments

#### Health parameters

##### Cardiac autonomic modulation

The assessment of cardiac autonomic modulation will be carried out using heart rate variability (HRV). For this evaluation, the subjects will be instructed that 12 h before carrying out the experimental protocol they do not use alcoholic beverages and/or stimulants (coffee and tea), so that there is no direct influence on cardiac autonomic behavior [18].

For HRV analysis, heart rate will be recorded beat by beat for 30 min in a room with controlled environment (temperature − 21 to 24° C; relative air humidity − 50 to 60%) [18]. For this analysis, a strap to capture electrical impulses will be positioned on the distal third of the participant’s sternum and will capture the heart’s electrical impulses and transmit such information through an electromagnetic field to a Polar brand heart rate receiver, model V800 (Polar Electro Oy, Kempele, Finland), which will be positioned on a of the participant’s wrists.

Participants will be placed in a supine position at rest and with spontaneous breathing. The series of RR intervals obtained will initially be submitted to digital filtering using the Kubios HRV® software (Biosignal Analysis and Medical Image Group, Department of Physics, University of Kuopio, Finland) with a median filter and and interval correction with a cubic spline interpolation method. After preprocessing the absence of artifacts or cardiac arrhythmias that may interfere with the HRV analysis will be observed through the visual analysis of the temporal series. In the present study, only series in which sinus beats are equal to or higher than 95% will be considered [19].

For HRV analysis, which will be carried out using linear methods (analyzed in the time and frequency domains) and non-linear methods, 1000 beats from the most stable period of the tracing will be used. For analyzes of linear and non-linear methods, the Kubios HRV® software (Biosignal Analysis and Medical Image Group, Department of Physics, University of Kuopio, Finland) and Visual Recurrence Analyzes (VRA) version 4.9 (Eugene Kononov, USA) will be used.

### Blood pressure and resting heart rate

Blood pressure will be assessed using an Omron automatic blood pressure measuring device (Model HEM-742, Japan). Before the first blood pressure measurement is assessed, individuals will remain seated at rest for five minutes, with their legs uncrossed, and will not be able to talk during this time. Two blood pressure measurements will be taken, and the average will determine the systolic and diastolic blood pressure value of the subject evaluated. These procedures are in line with the American Society of Cardiology [20].

### Body composition

General and abdominal obesity will be determined by weight and height measurements (which will determine the calculation of BMI in general obesity) and by waist circumference, respectively. The percentage of fat, fat-free mass and density and bone mineral content will be estimated using dual-energy x-ray absorptiometry (DEXA, Lunar Prodigy; General Electric Healthcare, Little Chalfont, Buckinghamshire, United Kingdom). The equipment’s own software will be used (GE Medical System Lunar, version 4.7).

### Handgrip strength

Handgrip strength will be assessed using a manual dynamometer in which two handgrip measurements will be taken on each arm. The highest value observed will be considered as an indicator of the strength level.

### Quality of life (QoL)

To assess QoL, the Medical Outcomes Study SF-36-Item Short Form Health Survey (SF-36) instrument will be used in the parents. The SF-36 is made up of 36 items that cover eight domains regarding QoL: functional capacity, physical limitations, body pain, general perception of health, vitality, social aspects, emotional limitations, mental health. The scores for this questionnaire range from 0 to 100 and the higher the score, the better the QoL for the analyzed domain [21].

The quality of life of children and adolescents will be assessed using the KIDSCREEN-52 questionnaire [22]. This instrument has 52 questions that analyze ten different domains (health and physical activity; feelings; emotional state; self-perception; autonomy; family environment; financial aspect; friends and social support; school environment and provocation / bullying) in pediatric populations. Based on the KIDSCREEN-52 scores, these values will be recoded to a scale of 0 to 100, where the higher the value, the better the undeveloped QoL domains. This instrument is validated for use with young Brazilians [23].

### Anxiety and depression

Symptoms of anxiety and depression will be analyzed using the Hospital Anxiety and Depression Scale (HADS). This instrument consists of 14 questions in total, half of which assess symptoms of anxiety and the other half of depression [24]. The score for each item varies from 0 to 3, providing a total score of 21 for each of the outcomes analyzed, and the higher the value, the worse the condition of the evaluated participant. This scale is also validated for use with young people [25].

### Stress

This aspect will be assessed in the parents and their children using the Brazilian version of the Perceived Stress Scale [26], validated by Siqueira Reis et al. [27] which consists of 14 items about the frequency of feelings and thoughts related to events and situations that occurred in the last month, seven items being negative (1, 2, 3, 8, 11, 12 and 14) and seven items being positive (4, 5, 6, 7, 9, 10, and 13). Each item is answered on a five-point Likert frequency scale, with 0 = never and 4 = very often. The stress score is constructed by adding the values answered in each of the items, with positive items being on an inverse scale (0 = very often and 4 = never). The total score varies from 0 to 56 points and the higher the score, the higher the stress level.

### Comorbidities

Self-reported morbidities will be assessed by the following question: - Have you ever been diagnosed by a doctor or are currently taking medication for a health problem: ( ) Yes; ( ) No;

If the answer is positive, you will be asked which morbidity is in question among a series of morbidities (hypertension, diabetes, high cholesterol, triglycerides).

### Musculoskeletal symptoms

The presence of musculoskeletal symptoms will be assessed using a questionnaire developed by Kuorinka et al. [28]. This instrument assesses some musculoskeletal symptoms such as the presence of pain, tingling or numbness in different parts of the body (neck, shoulder, upper back, elbows, wrists/hands, lower back, hip/thigh, knees and ankles/feet). It is noteworthy that this instrument is also validated for use with children and adolescents [29]. The intensity of pain and how long the participant has felt pain will also be assessed on a scale of zero to ten.

### Lifestyle habits

#### Intensity of physical activity

The different intensities of physical activity will be measured objectively through the Actigraph GT3X accelerometer (ActiGraph, LLC, Pensacola, FL, USA), which is a light, portable, small, easy-to-use instrument designed to be positioned on the subject to record movements in the three orthogonal planes: vertical, horizontal anteroposterior and mediolateral. The accelerometers will be positioned laterally at waist height for those evaluated, who will remain with the equipment for four full days (three weekdays and at least one weekend day) [30]. Participants will be instructed to use the equipment throughout the day, removing it only when there is contact with water (taking a shower or swimming, for example) and during sleeping hours.

The ActiLife 6 program (ActiGraph, LLC, Pensacola, FL, USA) will be used to clean the data. Each data sample, determined by counts, will be summarized considering a specific time interval, this specific interval is designated as epoch, and lasts 60 s. The 60-second period was selected because it is the closest to the low-intensity, long-duration activity pattern [31]. Consecutive hours of zero counts and days with less than 10 h of monitoring will be excluded [32].

We will use the cutoff point recommended by Troiano et al. [30] to analyze the different intensities of physical activity. Light physical activity will be defined as 101–2019 counts per minute, moderate physical activity will be defined as counts between 2020 and 5998, vigorous physical activity will be defined as counts greater than ≥ 5999 cpm counts per minute. Activities below 100 counts per minute will be considered time in sedentary behavior.

Children and adolescents will be classified according to the cutoff points recommended by Evenson et al. [33], light physical activity will be considered 101–2295 counts per minute, moderate physical activity will be defined as 2296–4011 counts per minute and vigorous physical activity ≥ 4012 counts per minute. Activities below 100 counts per minute will be considered sedentary behavior.

### Physical activity performed in different domains

Physical activity in different domains will be assessed using the questionnaire by Baecke et al. [34], validated for adults [35] and young Brazilians [36]. Baecke’s questionnaire consists of 16 questions in which the last twelve months are considered, evaluating occupational physical activities (work in the case of adults) and at school (in the case of children and adolescents), sports practice and/or systematized exercises in the leisure and active commuting. In the work (or school) domain, issues relating to physical efforts carried out at work (or school) are investigated, such as: time spent sitting, standing and walking in the work environment (or school), carrying weights, whether the subject being assessed sweats a lot in the workplace (or school), work and how tired you feel after a day of work (or school). In the leisure domain, leisure-time physical activity is considered, such as exercising at the gym or playing sports. In this domain, the intensity of the physical effort of these activities (light, moderate, vigorous), the number of hours per week in which these activities are practiced and how long this physical activity has been practiced (< 1 month, 1–3 months, 4–6 months, 7–9 months, > 9 months) will be evaluated. In the field of active commuting, different physical activities are considered for this purpose, especially considering the amount of time spent walking to the mall, market or work. The time spent cycling is also considered.

In the end, this instrument offers a dimensionless score for each of the domains and the sum of the scores for the three domains also determines the total amount of physical activity practiced by each of the individuals evaluated.

### Sedentary behavior

To assess sedentary behavior, participants (parents and children) will answer the Sedentary Behavior Questionnaire [37]. It includes the domains of sedentary behavior (watching TV, playing video games and computer, sitting listening to music, sitting talking or typing on the mobile phone, working on the computer or bureaucratic tasks {e.g. answering emails}, playing a musical instrument, doing construction work art or crafts, sitting in a car, bus, or train) during the week and on weekends. The time spent using social media (Instagram, WhatsApp, Twitter, Facebook, etc.) will also be taken into account.

### Sleep quality

The Mini-sleep Questionnaire [38] will be used in the present study to evaluate the quality of sleep of parents and their children. This instrument is validated for the Portuguese language [39] and has 10 questions with seven possible answer options (never = 1, very rarely = 2, rarely = 3, sometimes = 4, often = 5, very often = 6 and always = 7) and provides a dimensionless score (higher score, worse sleep quality). The score for this instrument ranges from 0 to 70, with worse sleep quality being identified in higher scores.

The amount of hours of sleep will be assessed by the number of hours slept by the participant on a typical night.

### Eating habits

Eating habits will be verified through the frequency of weekly consumption of different foods based on the work of Block et al. [40]. The consumption of foods such as vegetables, fruits, cereals, dairy products, fried foods, sodium and sugary drinks (soft drinks), as well as stimulant (energy) and caffeinated drinks will be analyzed. They will be characterized by high consumption of these subject foods with consumption equal to or greater than five times a week. Individuals who are classified as consuming vegetables, fruits, cereals and dairy products with a weekly frequency of less than five times a week and/or are classified as having an intake of sodium, fried foods and sugary drinks more frequently than five times a week will be classified as having inadequate food.

### Smoking and alcohol consumption

Smoking will be assessed using the following question: Do you smoke, have you smoked, or have you never smoked? If you smoke, how many cigarettes do you usually smoke per day, considering the last month? If you have smoked, how long have you smoked and how long have you stopped smoking?”

Exposure to tobacco among non-smokers and ex-smokers will be assessed by the following question: “Do you have close contact with any smokers? Yes or no?”

Alcohol consumption will be assessed by the weekly frequency of alcohol intake and the number of doses consumed. Adults who reported drinking alcoholic beverages with a frequency equal to or greater than 1–2 days a week with an intake of 1–2 drinks per day will be considered to have high alcohol consumption (each dose will correspond to 250 ml for beer and 40 ml for distilled beverages).

#### Counfounders

Sociodemographic variables of age, sex, ethnicity and socioeconomic status will be considered as covariates.

### Socioeconomic level

In determining the economic condition, the “Brazilian Economic Classification Criteria” established in 2022 by the Brazilian Association of Research Companies - ABEP [41] will be used. The questionnaire will be completed through a face-to-face interview and the level of education, presence and quantity of certain rooms and goods in the analyzed household will be considered. The source of the water will be questioned whether it comes from a well or spring, the general distribution network or other means, and whether the street of the household is asphalted/paved or made of dirt/gravel. The level of education of the head of the family will be questioned (considering the head of the family to be the one who contributes the largest part of the family income).

**Fig. 1:**
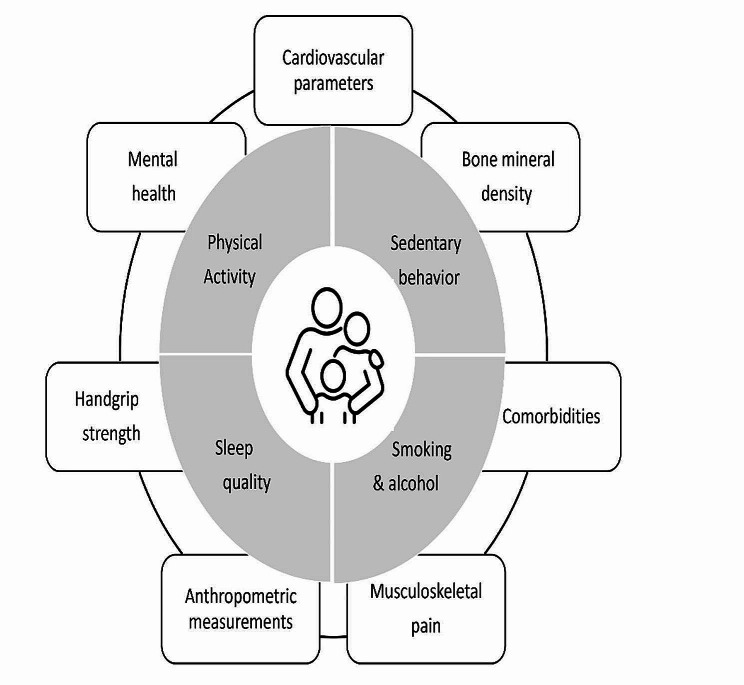
Infographic of the assessments to be carried out in this study

### Statistical analysis

The sample characterization variables will be expressed as mean and standard deviation if the data are parametric and median and interquartile range if they are not parametric. The association between lifestyle habits, as well as direct health measures carried out between parents and children, will be analyzed using Linear Regression adjusted by age of adolescents and their parents, socioeconomic status, parents’ education. In the case of using categorical analyses, Binary Logistic Regression or Poisson (with robust variance adjustment) will be used. The statistical significance to be used will be 5% and the confidence interval will be 95%. The statistical package used will be SPSS version 25.0.

## Discussion

This research project will aim to verify the influence of parents’ health characteristics and lifestyle habits on their children. To this end, children and adolescents aged 6–17 and their parents will be assessed. Such information will be important, since lifestyle habits developed in childhood and adolescence tend to persist into adulthood [1, 3].

The family environment tends to guide the lifestyle habits of young people, and the current study will monitor participating families longitudinally. Among the important lifestyle habits to be monitored, we highlight physical activity. Christofaro et al. [16], in a study that evaluated the relationship between physical activity carried out by parents currently and also in the parents’ own childhood and adolescence, observed that the practice of physical activity among parents was positively associated with the practice of physical activity in their children.

In addition to the lifestyle habits between parents and their children we will also explore the possible role that physical activity in the associations. A main novelty will be to consider the possible role of physical activity in its different domains, analyzing occupational physical activity (work or housework for parents and physical activity at school for children), leisure-time sports and commuting actives. This project will also consider the role of different intensities of physical activity (light, moderate and vigorous) in the associations of lifestyle habits of parents and children.

Children, adolescents and active adults are less likely to be affected by cardiovascular diseases, for example, which are responsible for the highest causes of death in the world [20]. In the short term, this study could provide important information about the family environment in which health promotion strategies can be created. One of the answers we are aiming to obtain is if different types of physical activity and different types of intensity will have the same role between the associations of parents’ and children’s habits, or it will be different. With these estimates, for example, it will be possible to identify where health promotion actions should be prioritized for the population investigated.

### Potential challenges

Like all longitudinal studies, which follow participants for a long period, there is naturally sample loss due to a series of factors, such as withdrawal from participating in the study, moving to another city, among others. However, some strategies were designed to mitigate sample losses as: (1) Will be to provided a didactic report with all the assessments carried out, indicating to the participants all the results of the health parameters carried out; (2) We will try to maintain contact via social media so that rapprochement with participants always occurs.

### Expected results

This study can be considered innovative based on its objectives. As initial positive aspects, we highlight the longitudinal design, the high number of subjects that will be evaluated and the performance of direct measurements with a high degree of sensitivity such as cardiac autonomic modulation (through HRV analysis), bone health being assessed by Radiological Absorptiometry Dual Energy (DEXA) and physical activity (to be assessed using accelerometry).

We believe that the results obtained in this research will allow improvements in the areas of cardiovascular risk factor analysis and also objective and subjective measures of physical activity in the family environment. It is expected that such results can contribute to the development of health promotion actions and where these efforts should be invested.

## Data Availability

No datasets were generated or analysed during the current study.

## Abbreviations

ABEP: Brazilian Association of Research Companies
BMI: Body mass index
CRF: Cardiovascular risk factors
DEXA: Radiological Absorptiometry Dual Energy
HRV: Heart rate variability
QoL: Quality of life
